# 

**Published:** 1851-06

**Authors:** 


### CONTENTS

### OF NO. VI.

---

### ORIGINAL COMMUNICATIONS.

### ESSAYS AND CASES.

ART. I.—On Carbonagogue Medicines. By LEWIS ROGERS, M. D., Professor of Materia Medica and Therapeutics, in the Medical Department of the University of Louisville. 461
ART. II.—Aristotle and Theophrastus. Translated from the French of M. Cap. By W. H. COBB, M. D., of Louisville, Ky. - - - - - - - - - 510

### SELECTIONS FROM FOREIGN AND HOME JOURNALS.

Results of the Use of Chloroform in 9000 cases at St. Bartholomew's Hospital. - - - - - - - - - 520
Value of a Chemical and Microscopic Examination of the Urine as a means of Diagnosis in Renal Diseases. - - 523
Vaccination as a Preventive of Small-pox. - - - - - 524
On the Treatment of Itch. - - - - - - - 526
Femoral Aneurism cured by Compression. - - - - - 527
Two Cases of Popliteal Aneurism successfully treated by Compression. - - - - - - - - - - 529
Treatment of Abscesses connected with Caries of Bones. - 533
Medical Society of London. - - - - - - - 534
Pathological Society of London. - - - - - - 538
Rheumatic Fever treated by Acetate of Potash. - - - 540

### ORIGINAL INTELLIGENCE.

University of Louisville. - - - - - - - - - 545
An Improved Microscope. - - - - - - - - - 546
Carbonagogue Medication. - - - - - - - - 548
Dr. E. D. Fenner. - - - - - - - - - - 548
Professor Silliman. - - - - - - - - - - 548

